# Comprehensive screening of target molecules by next-generation sequencing in patients with malignant solid tumors: guiding entry into phase I clinical trials

**DOI:** 10.1186/s12943-016-0553-z

**Published:** 2016-11-16

**Authors:** Yuko Tanabe, Hitoshi Ichikawa, Takashi Kohno, Hiroshi Yoshida, Takashi Kubo, Mamoru Kato, Satoru Iwasa, Atsushi Ochiai, Noboru Yamamoto, Yasuhiro Fujiwara, Kenji Tamura

**Affiliations:** 1 Department of Experimental Therapeutics, Exploratory Oncology Research & Clinical Center, National Cancer Center, 5-1-1, Tsukiji, Chuo-ku, Tokyo, 104-0045 Japan; 2 Department of Breast and Medical Oncology, National Cancer Center Hospital, 5-1-1, Tsukiji, Chuo-ku, Tokyo, 104-0045 Japan; 3 Division of Translational Research, Exploratory Oncology Research & Clinical Center, National Cancer Center, 5-1-1, Tsukiji, Chuo-ku, Tokyo, 104-0045 Japan; 4 Department of Clinical Genomics, National Cancer Center Research Institute, 5-1-1, Tsukiji, Chuo-ku, Tokyo, 104-0045 Japan; 5 Department of Pathology and Clinical Laboratories, National Cancer Center Hospital, 5-1-1, Tsukiji, Chuo-ku, Tokyo, 104-0045 Japan; 6 Department of Bioinformatics, National Cancer Center Research Institute, 5-1-1, Tsukiji, Chuo-ku, Tokyo, 104-0045 Japan; 7 Department of Medical Oncology and Translational Research, Graduate School of Medical Sciences, Kumamoto University, 1-1-1 Honjyo, Chuo-ku, Kumamoto, 104-0045 Japan

**Keywords:** Next-generation sequencing, Phase I trial, Molecular pre-screening, Molecular targeted therapy, Driver mutation

## Abstract

It is still controversial whether comprehensive genome screening of target molecules by next generation sequencing (NGS) is needed to increase clinical efficacy of investigational drugs or accelerate drug development, although several studies are being carried out. Therefore, we performed a prospective study to evaluate the feasibility of comprehensive gene screening in this setting. Our findings indicate that actionable alterations were identified in 45% of the analyzed patients, most frequently in those with breast cancer. Common actionable alterations were found in *PIK3CA* mutation, *BRCA2* mutation, *ERBB2* amplification, and *CCND1* amplification. In total, 22% of the analyzed patients could be entered into phase I clinical trials, and 8% of them were treated with “matched” drugs. Among patients who received matched therapies, response and disease control rates were 33 and 78%, respectively. On the other hand, in the patients who received “non-matched” therapy, the objective response rate was 6%. We believe this data indicates that NGS-based molecular pre-screening is a potent platform for use before patient entry into phase I trials.

**Electronic supplementary material:**

The online version of this article (doi:10.1186/s12943-016-0553-z) contains supplementary material, which is available to authorized users.

## Findings

### Background

Recent molecular characterization studies showed that many alterations occurred in oncogenes or tumor suppressor genes that might become therapeutic targets, for instance, *PIK3CA* [1], *AKT1* [2], *FGFR1* [3–5], and *BRCA1/2* [6]*.* Screening for such targetable genetic alterations will aid in identifying subpopulations of patients who will benefit from specific treatments.

In phase I trials, information on genomic alterations in tumors is quite helpful to allow each patient entry to a suitable clinical trial, in which the molecular targeted drug is theoretically matched to the alterations. If we were to find super-responders in these trials, the genomic alterations would be recognized as specific biomarkers to predict the response to the investigational drug.

Therefore, we conducted a prospective cohort study to investigate the feasibility of NGS-based pre-screening to identify genomic alterations in patients considering entry into phase I clinical trials (Additional file 1: Supplementary Methods) [7–15]. We named this study the “Trial of Oncopanel for Introduction into Clinical Study-Phase 1 (TOPICS-1).”

## Results

### Registration and sequencing

**Fig. 1:**
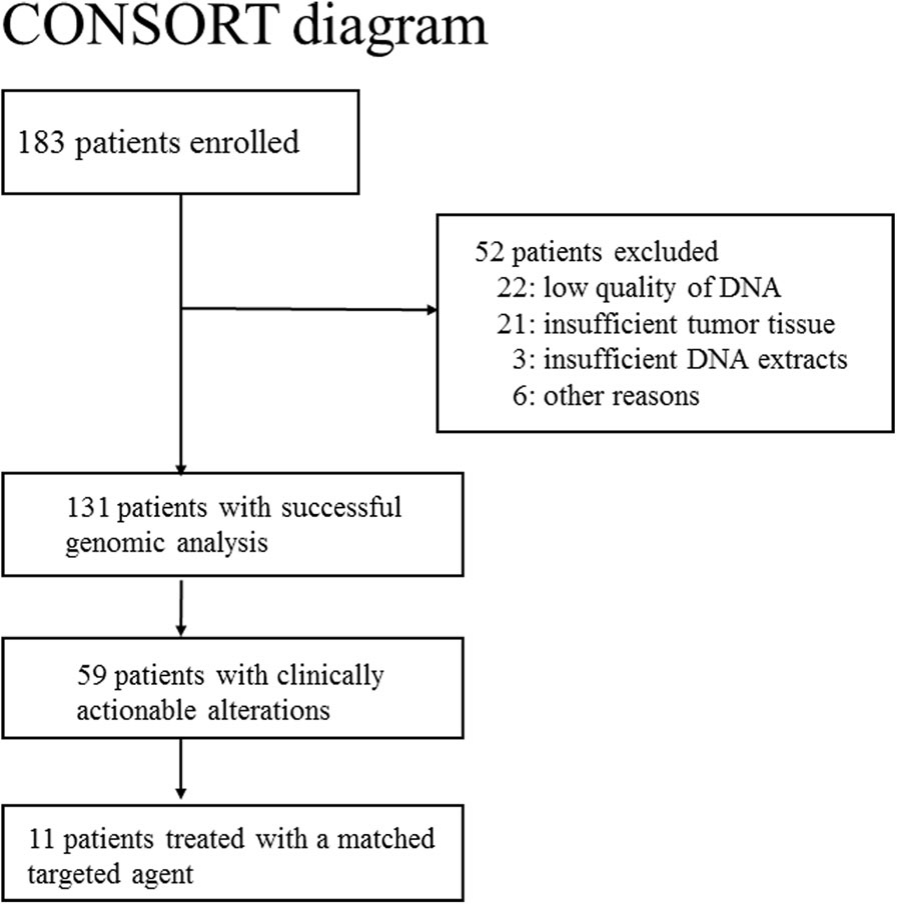
Registered and analyzed tumors in the TOPICS-1 study. The diagnoses of patients who underwent genomic testing. Each diagnosis included a variety of different histologic subtypes

From July 2013 to October 2014, 183 patients were recruited for the study (Fig. 1 and Additional file 2: Table S1). Fifty-two patients were omitted from sequencing analysis. The major reasons were low-quality DNA (22 patients) and insufficient tumor tissue (21 patients). Less common reasons included insufficient DNA quantities (3 patients) and other factors (6 patients) (Additional file 2: Table S1). The success rates were higher in surgical samples (93/125, 74.4%) than in biopsy samples (38/58, 65.5%). As a result sequencing was performed in 131 patients. The types of cancer were as follows: 35 breast (27%), 35 gastric (27%), 21 ovarian (16%), 12 lung (9%), 8 bile duct (6%), 5 cervical (4%), 4 thymic (3%), 3 endometrial (2%), 3 colon (2%), 2 sarcomas (2%), and 3 others (2%) (Fig. 1). Thirty (79%) of 38 biopsied samples and 83 (89%) of 93 surgical samples were primary tumor lesions. The other biopsy samples were derived from metastases in the liver (4 patients), lymph nodes (2 patients), and skin (2 patients) (Additional file 2: Table S1).

### Detected genomic alterations

**Fig. 2:**
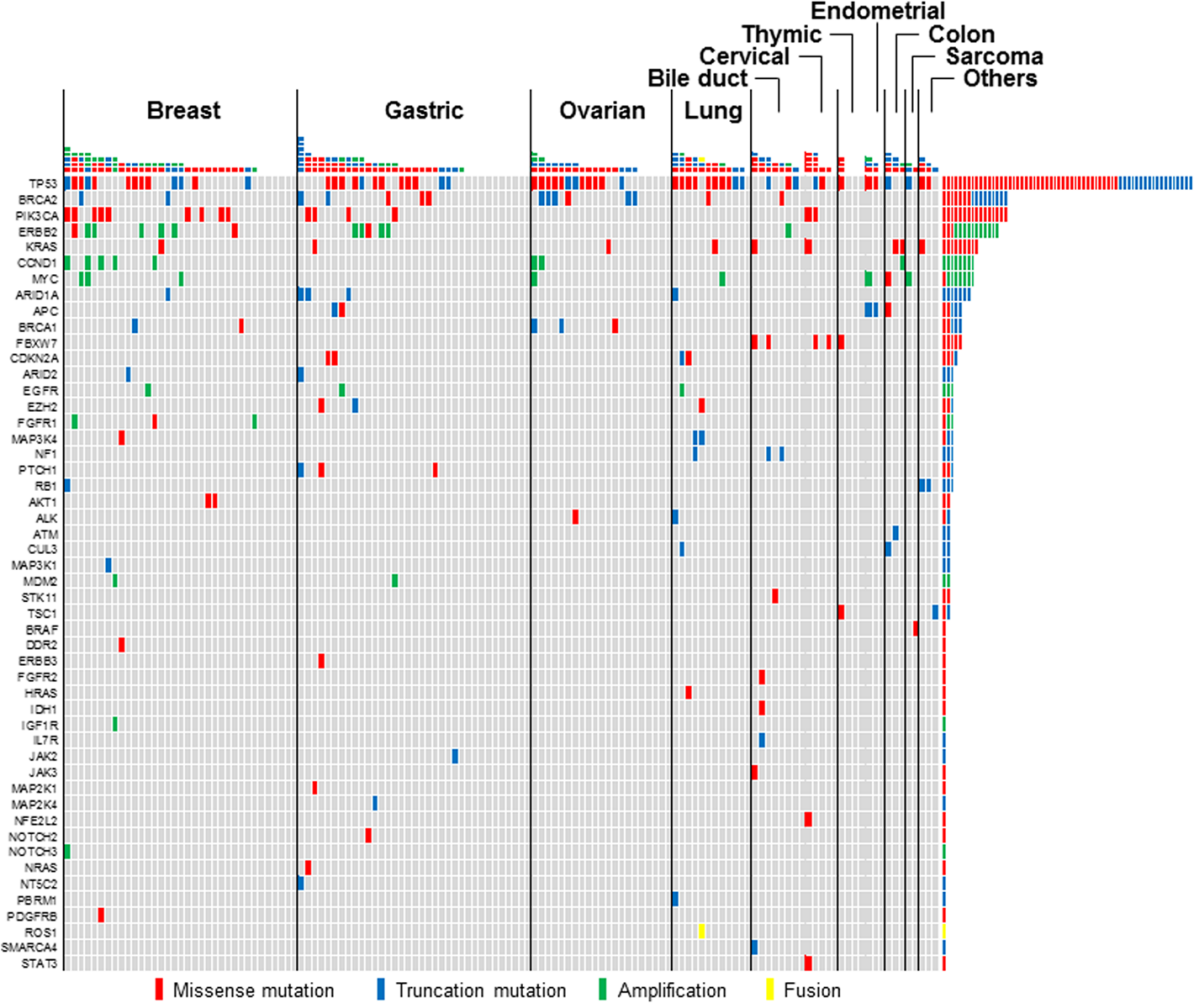
Summary of identified genomic alterations. The mutations, amplifications and fusions observed and reported in the study

We identified 1.42 mutations (Additional file 3: Table S5) and 0.26 amplifications (Additional file 4: Table S6) per patient. In addition, a fusion gene (*CD74-ROS1*) was found in 1 patient. Of the 131 patients, 104 (79%) had at least 1 genomic alteration, and 59 (45%) had at least 1 actionable genomic alteration (Additional file 5: Table S7). The most frequent genomic alterations were *TP53* mutations (59 [46%] of 131 patients), *PIK3CA* mutations (15 patients [11%]), *ERBB2* amplifications and mutations (12 patients [9%]), *BRCA2* mutations (8 patients [6%]), *CCND1* amplifications (8 patients [6%]), *KRAS* mutations (8 patients [6%]), *MYC* amplifications (7 patients [5%]), and *ARID1A* mutations (5 patients [4%]) (Fig. 2). Among them, we considered *PIK3CA* mutations, *ERBB2* amplifications and mutations, *BRCA2* mutations, and *CCND1* amplifications as actionable. Other actionable alterations were less common, including *BRCA1* mutations (4 patients [2%]), *EGFR* amplifications (2 patients [2%]), *AKT1* mutations (2 patients [2%]), *MDM2* amplifications (2 patients [2%]), *FGFR1* amplifications (2 patients [2%]), and a *ROS1* fusion (1 patient [1%]).

### Entry into phase I clinical trials

By June 2015, 29 (22%) of the 131 patients who underwent the sequencing test had entered phase I trials. Therefore, the primary endpoint, the inclusion of 25% patients in phase I trials, was not met. Seventeen different regimens were used in the 29 patients who entered phase I trials. Eleven (8% of the 131 patients) entered phase I trials of targeted therapies that matched their genomic alterations (Table 1). Forty-two of 59 patients who had actionable alterations could not enter accessible phase I trials because of ongoing standard treatment (18 patients), disease progression (18 patients), the patients’ wishes (4 patients), or no available matched phase I trials that targeted their actionable alterations (2 patients).

**Table 1:** Characteristic of patient entering into Phase I with matched therapy

No.	Cancer type	Molecular alteration	Matched therapy	Response	Progression free survival (month)
1	Bile duct	*FGFR2*	FGFR inhibitor	PD	0.95
2	Cervical	*PIK3CA* E542K	PI3K inhibitor	NE	2.53
3	Liver	*TSC1*	mTOR inhibitor	NE	0.82
4	Breast	*BRCA1*	PARP inhibitor	PR	8.09
5	Peritoneal	*BRCA1*	PARP inhibitor	SD	7.17
6	Cervical	*PIK3CA* E542K	AKT inhibitor	PD	0.72
7	Breast	*PIK3CA* H1047R	PI3K inhibitor	PR	6.18
8	Breast	*PIK3CA* E545K	PI3K inhibitor	SD	2.80
9	Breast	*PIK3CA* E545V	PI3K inhibitor	SD	5.72
10	Breast	*AKT1* E17K	AKT inhibitor	PR	14.1
11	Breast	*BRCA1*	PARP inhibitor	SD	5.53

*PD* progression disease, *SD* stable disease, *PR* partial response, *NE* not evaluable

### Matched therapies and response

Seven different regimens were used in the 11 patients who received matched therapy. Two patients could not be assessed for antitumor activity. We evaluated response in the 9 assessable patients. Of them, 3 showed response and 4 had stable disease (Table 1). Partial response (PR) was seen in 3 patients, all responders had breast cancer. Case 1 was an ER-positive breast cancer patient with a *PIK3CA* H1047R mutation. She was treated with 5 kinds of hormonal therapy and 3 kinds of chemotherapy containing anthracycline and capecitabine after first relapse, and then received PI3K inhibitor treatment. PR began 60 days after first administration and lasted for 16 months. Case 2 was an ER-positive breast cancer patient with an *AKT1* E17K mutation. She was treated with 6 kinds of hormonal therapy and 3 kinds of chemotherapy containing anthracycline and taxane, and then received AKT inhibitor treatment. PR began 60 days after first administration and lasted for 27 months. Case 3 was a triple-negative breast cancer patient with a *BRCA1* truncation mutation. She experienced her first relapse after adjuvant chemotherapy containing anthracycline and taxane and then received combination therapy with a PARP inhibitor and eribulin. PR began 60 days after first administration and lasted for 6 months. The response and disease control rates were 33 and 78%, respectively. In contrast, the 18 patients who received non-matched therapy had response and disease control rates of only 6 and 56%, respectively. Moreover, we assessed progression free survival (PFS) (Table 1). Median PFS of the patients with matched therapy was longer than those with non-matched therapy (5.5 months, 95% CI; 2.1 to 9.0 vs. 1.9 months, 95% CI; 0.5 to 3.2) (Additional file 6: Figure S1).

Several trials, including SAFIR01 [NCT01414933] [7], NCI-MATCH [NCT02465060], MOSCATO01 and −02 trials [NCT01566019], SHIVA [NCT01771458] [16], PROFILER [NCT01774409], the EORTC SPECTA initiatives, are carried out to investigate the feasibility and utility of NGS-based screening. However, it is controversial for phase I trials, especially.

We demonstrate the feasibility of in-house, gene panel–based NGS screening for entry into phase I clinical trials for anti-cancer drugs. One of the distinctive features of this study is the customized assay design. Considering the flexibility of target genes, we adopted a custom gene panel consisting of 90 genes for mutations and amplifications and 10 genes for fusions (Additional file 7: Table S4). The analytical accuracy of this in-house system was validated. The second distinctive feature of this study is the use of formalin-fixed paraffin-embedded (FFPE) samples, which are easily available in clinical practice. The use of FFPE samples for sequencing creates the opportunity to characterize cancer-relevant genes even in cases where tissue retrieval is difficult. The quality of FFPE samples was related to fixation time and storage duration. To ensure stable sequencing, we changed the DNA amounts used for library preparation in response to the quality of the extracted DNA (Additional file 8: Table S2).

Genomic analysis was performed in 72% of the enrolled patients, and enabled matching of therapy in 8% of the patients in whom sequencing was performed. Twenty-two percent of the analyzed patients entered phase I trials after the sequencing test, although the primary endpoint was not met. This result was affected by patients’ performance status and the numbers of accessible phase I trials. However, it was feasible for the candidate patients to entry to phase I trials based on sequencing results. Moreover, genomic analyses led to PR in 33% and disease control in 78% of the patients who received matched therapy. The success rate of receiving matched therapy was consistent with other report [17]. On average, phase I trials show response rates between 5 and 10% [18–21]. In addition, in this study the response and disease control rates of genomic alteration–matched therapies were higher than those of non-matched therapies (33% versus 6%, and 78% versus 56%, respectively). Median PFS of the patients with matched therapy was longer than those with non-matched therapy (5.5 months, 95% CI; 2.1 to 9.0 vs. 1.9 months, 95% CI; 0.5 to 3.2). These results suggest the clinical utility of the sequencing test. The value of the sequencing test should increase if more predictive markers are defined or more novel targeted therapies are developed.

“Actionable genomic alterations” are a moving target. The evidence level of these alterations will probably change in the coming years as experimental agents move through the developmental pipeline. Many of the tumors that we tested harbored more than one potentially actionable alteration, but few treatment algorithms existed to stratify treatment options for these cases. In our original gene panel, target genes can be flexibly changed responding to the needs of the study (customized panel).

The current study involved 9 tumor types. The ratio of patients who were able to receive matched therapy was higher in breast cancer patients than those with other cancer types, and objective responses were observed only in breast cancer patients. The high efficacy in this population might be due to the higher frequency of driver mutations in breast cancer than in other tumor types (Additional file 9: Table S3). Moreover, it might be helpful that breast cancer is less aggressive and the tumor tissue is easy to access. Given our results, it is likely that the clinical utility of molecular prescreening differs among tumor types, and organ-specific screening might be useful.

Regarding future directions, we first need to determine the utility of small, organ-specific gene panels compared with the present pan-cancer gene panel. Second, we need to reconsider the timing of sequencing tests, such as perioperatively or at first recurrence. Third, to improve the accessibility of target drugs we need to construct a global social networking system that will allow patients to enter clinical trials.

## Conclusions

This report showed that the NGS-based molecular screening was feasible in clinical setting and would be potentially useful for selecting adequate patients for entry into clinical studies. Since most recent phase I trials have tried to identify early signals regarding the efficacy of targeted agents, there is a strong rationale for proposing molecular selection for patients eligible for these studies.

## Additional files

Additional file 1: Supplementary Methods. (DOCX 17 kb)
Additional file 2: Table S1. List of patients registered into the study. (XLSX 21 kb)
Additional file 3: Table S5. List of identified mutations after SNP elimination. (XLSX 27 kb)
Additional file 4: Table S6. List of identified amplifications. (XLSX 10 kb)
Additional file 5: Table S7. List of actionable genomic alterations. (XLSX 10 kb)
Additional file 6: Figure S1. Progression free survival of matched and non-matched therapy. (TIF 111 kb)
Additional file 7: Table S4. Gene list of NCC oncopanel ver. 2. (XLSX 10 kb)
Additional file 8: Table S2. DNA quantities used for sequencing library preparation. (XLSX 9 kb)
Additional file 9: Table S3. Actionable genomic alterations in histological tumor types. (XLSX 10 kb)
