# Supplementary material for: Comprehensive screening of target molecules by next-generation sequencing in patients with malignant solid tumors: guiding entry into phase I clinical trials
Source: Mol Cancer. 2016 Nov 16;15:73. doi: 10.1186/s12943-016-0553-z (PMC5112718; doi:10.1186/s12943-016-0553-z)
Supplement: Additional file 1: — Supplementary Methods. (DOCX 17 kb) [file 12943_2016_553_MOESM1_ESM.docx]

**Methods**

**study design**

The inclusion criteria were metastatic solid tumors with archived tissues, patient age of >20 years, performance status of 0 or 1, and stable disease. After patients provided written informed consent, the samples for sequencing were selected from the archived tumor tissues stored in the National Cancer Center Biobank and sent to the laboratory. The sequencing was performed as described below, and the results were discussed in conferences held twice a month by a multidisciplinary team including biologists, genome researchers, bioinformaticians, pathologists, and clinicians. Decisions regarding treatment were made during these conferences. Patients could enter the phase I trial on the basis of these decisions at the time of disease progression after standard treatment. The study was approved by the local ethics committee. All study participants provided informed consent to participate in this study and to publish the data of this study.

**samples and DNA preparation**

We used FFPE tumor tissues as starting materials for sequencing analysis. Genomic DNA was prepared with a QIAamp DNA FFPE tissue kit (QIAGEN, Hilden, Germany), and quantified using a Qubit dsDNA BR assay kit (Thermo Fisher Scientific, MA, USA) and by quantitative PCR analysis. The ratio of PCR-amplifiable DNA to total dsDNA as a value indicating the DNA quality. When this quality value was 0.1 or more, we went on to sequencing (Table S2).

**targeted sequencing**

We designed an original gene panel (NCC oncopanel v2). This panel captured all coding exons of 90 genes and all reportedly translocated introns of 10 genes (Table S4). Sequencing libraries were prepared using SureSelect XT reagent (Agilent Technologies) and a KAPA Hyper Prep kit (KAPA Biosystems, MA, USA) (Table S2), and were analyzed on a MiSeq sequencer (Illumina, CA, USA).

**bioinformatics analysis**

We used an in-house program to detect mutations (single nucleotide variants (SNVs) and short insertions and deletions (indels)), gene amplifications, and gene fusions. Mapping of NGS reads to the human reference genome was performed by BWA [13] and BWA-SW [14]. All alterations detected by our program were checked by manual inspection on the Integrative Genomics Viewer (IGV) [15]. For annotation and single nucleotide polymorphism (SNP) elimination, we used ANNOVAR [16], COSMIC [17], 1000 Genomes [18], ESP6500 [19], Human Genetic Variation Database [20], and in-house Japanese germline SNP data. All identified mutations after SNP elimination and all identified amplifications were summarized in Table S5 and S6, respectively. COSMIC database–registered mutations in oncogenes were considered as potential activating mutations. Truncating mutations and COSMIC database–registered mutations in tumor suppressor genes were considered as inactivating mutations. Final decisions were made in conference by the multidisciplinary team.

**definition of actionable genomic alterations**

The actionable genomic alterations were defined as those predicted to confer sensitivity to either an approved targeted agent or an experimental targeted agent in clinical trials. Table S7 summarized the genes in which those actionable alterations were found in this study.

**evaluation of response**

To analyze the effect on patient outcomes, we used the Response Evaluation Criteria in Solid Tumors [RECIST], version 1.1 [21]. In this study, complete response (CR) and partial response (PR) were considered as objective responses, and CR, PR, and stable disease (SD) for more than 16 weeks were considered as disease control.

**statistical analysis**

The primary endpoint of this study was the proportion of patients who could be entered into phase I clinical trials after the sequencing, with a goal of at least 25%. The secondary endpoint was the proportion of screened patients treated with the matched molecular target drugs, with a goal of at least 10%. The other secondary endpoint was to assess the efficacy of the approach in terms of achieving tumor responses, and the reasons for any inability to perform genomic analyses. We planned in advance to prospectively recruit patients until the number of patients who had successfully completed genome sequencing reached 130. With the inclusion of 130 patients, the study would have more than 90% power with a one-sided, level 0.05 test, assuming that the molecular screening program could drive up to 25% of patients (*n* = 32) into phase I trials. With a proportion lower than 20%, we judged that the program would not be effective.
