# Supplementary figures and images for: Comprehensive screening of target molecules by next-generation sequencing in patients with malignant solid tumors: guiding entry into phase I clinical trials
Source: Mol Cancer. 2016 Nov 16;15:73. doi: 10.1186/s12943-016-0553-z (PMC5112718; doi:10.1186/s12943-016-0553-z)

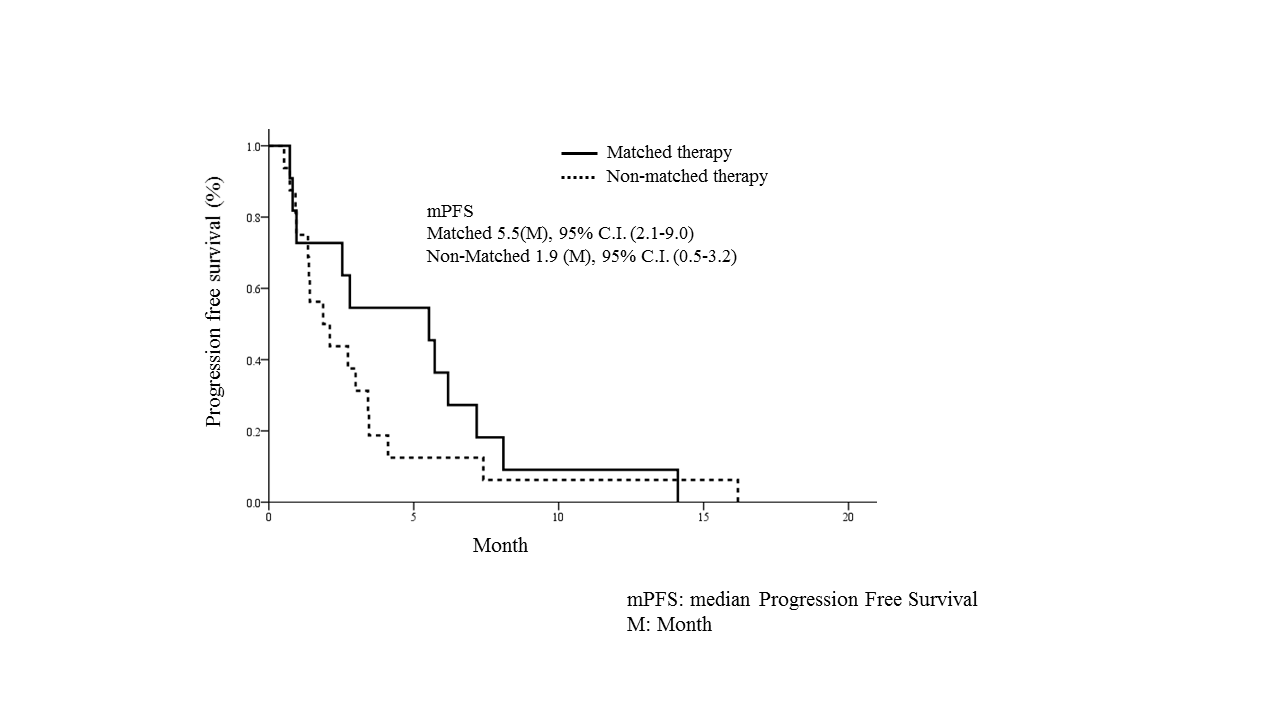

Supplement: Additional file 6: Figure S1. — Progression free survival of matched and non-matched therapy. (TIF 111 kb) [file 12943_2016_553_MOESM6_ESM.tif]
